# Whole genome sequence analysis reveals high genetic variation of newly isolated *Acidithiobacillus ferrooxidans* IO-2C

**DOI:** 10.1038/s41598-019-49213-x

**Published:** 2019-09-10

**Authors:** Anila Fariq, John C. Blazier, Azra Yasmin, Terry J. Gentry, Youjun Deng

**Affiliations:** 1 Microbiology & Biotechnology Research Lab, Department of Biotechnology, Fatima Jinnah Women University, Rawalpindi, 46000 Pakistan; 2 Department of Soil and Crop Sciences, Texas A&M University, College Station, TX 77843 USA; 3 Texas A&M Institute of Genome Sciences and Society, Texas A&M University, College Station, TX 77843 USA

**Keywords:** Genomics, Environmental microbiology

## Abstract

*Acidithiobacillus ferrooxidans*, a chemolithoautotrophic bacterium, is well known for its mineral oxidizing properties. The current study combines experimental and whole genome sequencing approaches to investigate an iron oxidizing, extreme acidophilic bacterium, *A*. *ferrooxidans* isolate (IO-2C) from an acid seep area near Carlos, TX, USA. Strain IO-2C was capable of oxidizing iron i.e. iron sulphate and iron ammonium sulphate yielding shwertmannite and jarosite minerals. Further, the bacterium’s genome was sequenced, assembled and annotated to study its general features, structure and functions. To determine genetic heterogeneity, it was compared with the genomes of other published *A*. *ferrooxidans* strains. Pan-genome analysis displayed low gene conservation and significant genetic diversity in *A*. *ferrooxidans* species comprising of 6926 protein coding sequences with 23.04% (1596) core genes, 46.13% (3195) unique and 30.82% (2135) accessory genes. Variant analysis showed >75,000 variants, 287 of them with a predicted high impact, in *A*. *ferrooxidans* IO-2C genome compared to the reference strain, resulting in abandonment of some important functional key genes. The genome contains numerous functional genes for iron and sulphur metabolism, nitrogen fixation, secondary metabolites, degradation of aromatic compounds, and multidrug and heavy metal resistance. This study demonstrated the bio-oxidation of iron by newly isolated *A*. *ferrooxidans* IO-2C under acidic conditions, which was further supported by genomic analysis. Genomic analysis of this strain provided valuable information about the complement of genes responsible for the utilization of iron and tolerance of other metals.

## Introduction

Diverse acidic environments with a wide range of acidity are found in natural and man-made settings on Earth. Areas with relatively low pH often contain high concentrations of sulphur or pyrite exposed to air. Oxidation of sulphur results in production of sulphuric acid and ferrous ion is oxidized to ferric. Acidophiles accelerate this oxidation process up to 106 times^1^. Though mechanisms of pH homeostasis in acidophiles are not fully understood, genome sequencing of acidophiles has revealed a number of processes microorganisms use to cope with low pH environments include impermeable cell membranes, organic acid degradation, cytoplasmic buffering and active proton extrusion^2^.

Acidithiobacilli are a group of sulphur-oxidizing acidophilic bacteria that exist in low pH environments. They are involved in acid mine drainage formation and have been used for the processing of different minerals. Seven species of Acidithiobacilli evident so far include *Acidithiobacillus thiooxidans*, *A*. *ferriphilus*, *A*. *albertensis*, *A*. *ferrooxidans*, *A*. *ferrivorans*, *A*. *ferridurans* and *A*. *caldus*^3^.

Whole genome sequencing of different species of Acidithiobacilli has provided new insights into their functions. A bulk of genes associated with carbon dioxide and dinitrogen fixation, pH resistance, oxidative stress, and heavy metal detoxification have been identified in whole genome sequence of *A*. *ferrooxidans* YQH-1^4^. Another study demonstrated sulphur oxidation in the extremophile *Acidithiobacillus thiooxidans* through whole-genome sequence analysis using a bioinformatic approach^5^. Functional predictions through genome analysis can be validated further using experimental approaches like Liljeqvist *et al*.^6^, who used transcriptional analysis to study the substrate regulation and identification of genes of psychrotolerant *Acidithiobacillus ferrivorans* during a bioleaching process. Although metabolic pathways for iron oxidation have been extensively studied, a little is known about the minerals produced during bioleaching process. Besides, only few genomes of *A*. *ferrooxidans* have been published, and no major report concerning their comparison or pan genome analysis has been published so far.

Areas around the Texas Municipal Power Agency’s (TMPA) Gibbons Creek lignite mine in east-central Texas, near Carlos, are rich with sulphur creeks/springs and natural acid seeps as a surface manifestation of the natural sulphur cycle. Sulphuric acid is generated when iron disulphide (pyrite) oxidizes and there are insufficient bases to neutralize it. Pyrite is common in lignite-bearing rocks because the anaerobic conditions favourable to the formation of lignite were also favourable to the chemical reduction of sulphate to sulphide. Mine-related acid seeps form in the same way that natural seeps do, except on an accelerated time scale^7^. The microflora responsible for the oxidation of pyrite in these seeps have yet to be explored. To this end, we have isolated the pyrite oxidizing bacterium i.e. *Acidithiobacillus ferrooxidans* IO-2C, from acid seep soil and investigated its iron oxidation potential. Mineralogy of the products formed as a result of bio-oxidation was also studied. Furthermore, whole genome analysis was performed to explore its genomic structure, properties and functional genes responsible for iron oxidation. Also, IO-2C genome was compared with other related species genomes to elucidate the degrees of genetic heterogeneity and gene conservation in *Acidithiobacillus ferrooxidans*.

## Results and Discussion

### Isolation and identification of strain

*Acidithiobacillus ferrooxidans* is abundant in acid rich, pyritic ores, and acid mine drainage related environments. Strain IO-2C was isolated on FeTSB medium containing 1% FeSO4 as yellowish-brown colonies at pH 2 and 25 °C after a week of incubation. Initial blastn search of 16S rRNA gene sequence in NCBI database showed highest similarity (99%) of isolate IO-2C with *Acidithiobacillus sp*. LLS-1 (Accession number: KT203930), a strain for which no genome assembly is available. Partial 16S nucleotide sequence was deposited in NCBI Genbank database under the accession number MH027513.

### Iron oxidation by *Acidithiobacillus ferrooxidans* IO-2C

**Figure 1:**
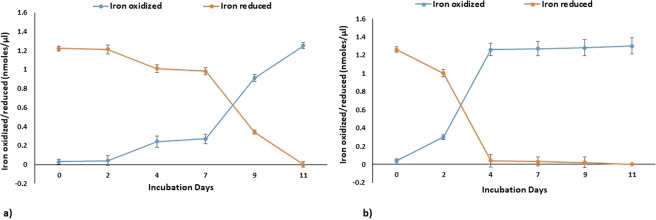
Bio-oxidation of (**a**) FeSO_4_ and (**b**) Fe(NH_4_)_2_SO_4_ by *Acidithiobacillus ferrooxidans* IO-2C.

**Figure 2:**
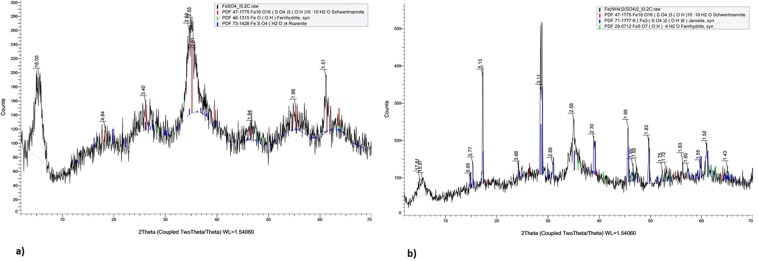
XRD spectra of iron oxides produced by *Acidithiobacillus ferrooxidans* IO-2C using (**a**) FeSO_4_ (**b**) Fe(NH_4_)_2_SO_4_ as substrates.

Acidophilic microbes possess the ability to alter the physical and chemical states of metals and form biominerals of industrial significance and also play a substantial role in biogeochemical cycling of metals^8^. *Acidithiobacillus ferrooxidans* possess the ability to oxidize solid substrates, like pyrite, aerobically. Oxidation of pyrite may occur outside the cell or in cell membrane. In acidic environments, such bacteria gain energy either by the oxidation of ferrous iron through reverse electron flow from Fe(II) to NADH or by oxidizing sulphur compounds, formate and hydrogen^9^. The oxidation kinetics and the iron oxide formed by *Acidithiobacillus ferrooxidans* IO-2C were affected by the companion cations in ferrous iron source. In case of 1% iron sulphate substrate, maximum oxidation of iron was observed after seven days of incubation. Rate of oxidation increased with increase in growth rate and incubation time (Fig. 1a). However, in case of 1% iron ammonium sulphate substrate, maximum oxidation of iron was recorded on fourth day of incubation and with the further increase in incubation time no significant oxidation was observed (Fig. 1b). More rapid iron oxidation was observed in case of iron ammonium sulphate as compared to iron sulphate which indicated that IO-2C strain may prefer ammonium-containing iron substrates to meet nitrogen needs and may possess a suite of genes for nitrogen metabolism working in tandem with iron and sulphur metabolism. Bio-minerals produced as a product of oxidation were analysed through FTIR (Supplementary Fig. 2), XRD (Fig. 2), and SEM (Supplementary Fig. 3) indicated the presence of shwertmannite, jarosite and minor quantities of ferrihydrite and rozenite as indicated by Nazari *et al*.^10^.

### Genome overview

**Figure 3:**
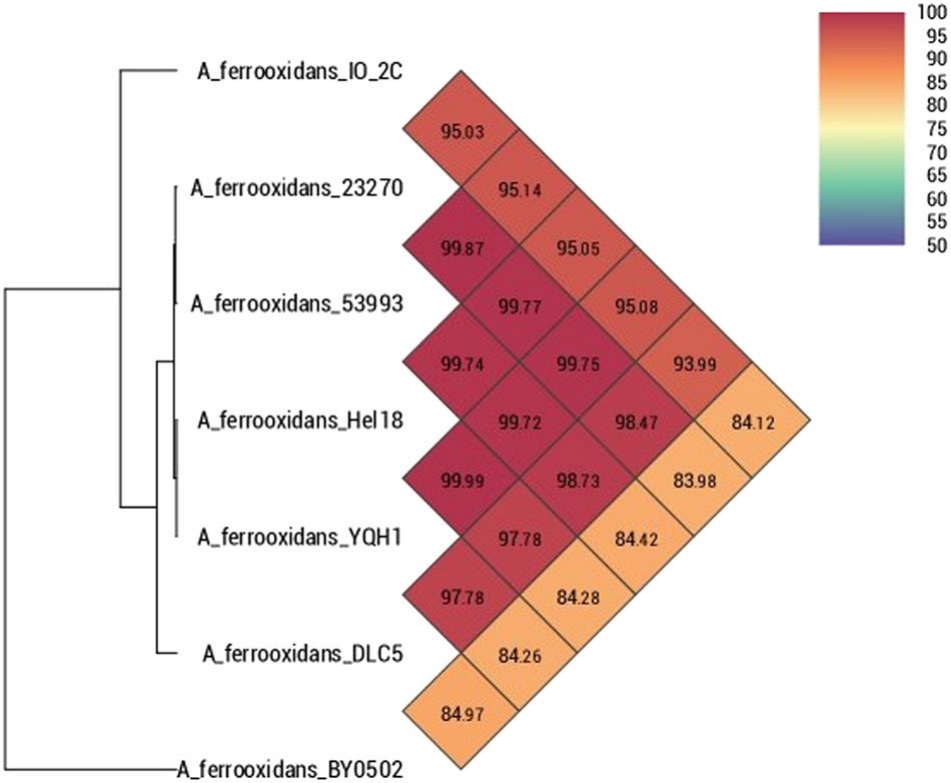
Heat map representing average pairwise nucleotide identity of *Acidithiobacillus ferrooxidans* IO-2C with other genomes of the same genus.

**Figure 4:**
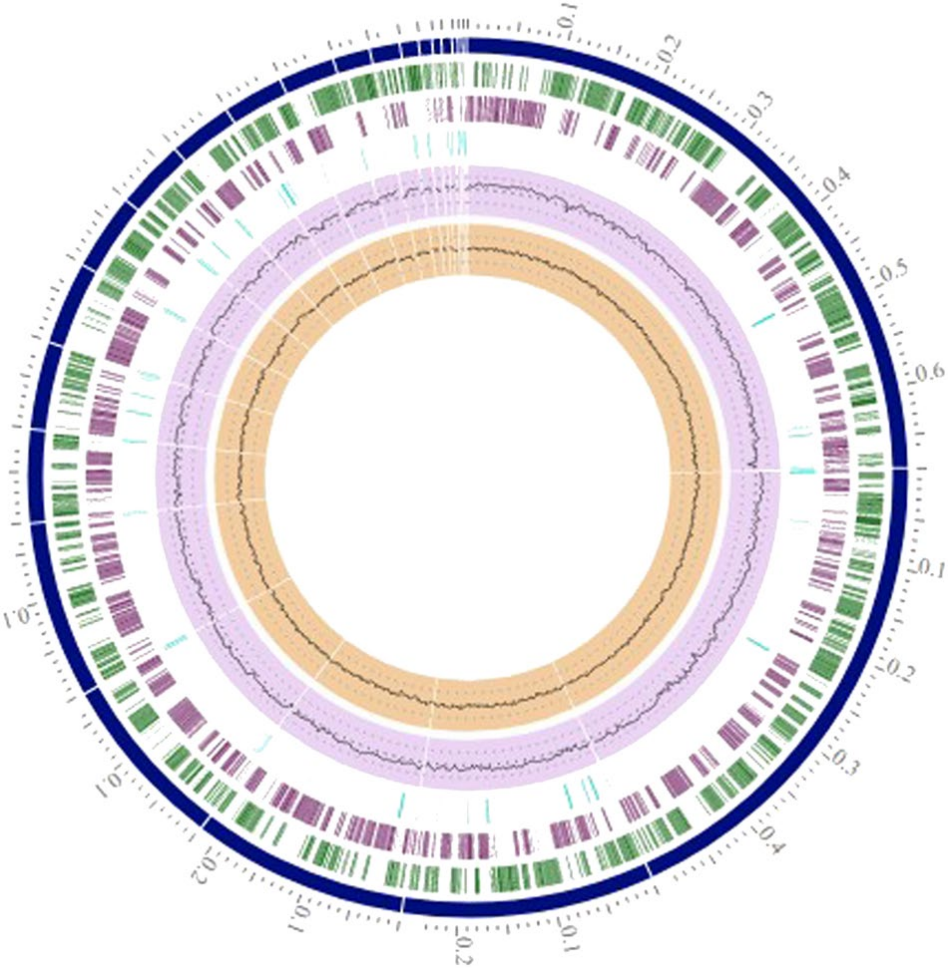
Circular genome map of *Acidithiobacillus ferrooxidans* IO-2C. Contigs represented in blue color; CDS-FWD in green; CDS-REV in purple; Non-CDS features in light blue; GC content in violet and GC skew in brown color.

The genome of *Acidithiobacillus ferrooxidans* IO-2C was sequenced to study its features and functions. The genome assembly comprised of 2,716,894 base pairs distributed in 23 contigs, with a G + C content of 58.73%. Average pairwise nucleotide identity obtained after comparison of our genome with other published *Acidithiobacillus ferrooxidans* genomes revealed maximum OrthoANI value of 95.14% with *Acidithiobacillus ferrooxidans* strain 53993 (Fig. 3). This relatively low pairwise nucleotide identity is attributed to high genetic heterogeneity in IO-2C genome which might be consequence of adaptation to hostile environmental conditions, i.e. pH stress or horizontal gene transfer over a long period of time^11^, which contributed to the large number of unique genes (292 genes including genes of unknown function and pseudogenes). Genome annotations revealed about 2, 927 protein coding sequences, 42 tRNAs, 5 rRNAs and 131 pseudogenes (Fig. 4). It contained numerous genes for iron and sulfur metabolism, aromatic compound degradation, stress response and metal resistance (Table 1). However, it lacks genes for motility and chemotaxis. PATRIC annotation of genome also depicted specialty genes including 2 for virulence factors, 5 drug targets and 9 for antibiotic resistance.

**Table 1:** List of selected genes identified in PGAP annotation of IO-2C strain showing genes for iron and sulphur metabolism, and metal and drug resistance.

Serial No.	Gene Name	Nucleotide position	Gene length	Locus tags
1	Arsenical resistance protein ArsH	4219..4485	266	C3R74_03365
2	Arsenical resistance operon transcriptional repressor ArsD	939..1301	362	C3R74_12890
3	Mercury(II) reductase	307817..309505	1688	C3R74_05010
4	Multidrug resistance efflux pump	176616..177785	1169	C3R74_08275
5	Drug resistance transporter, EmrB/QacA subfamily	113636..115099	1463	C3R74_07980
6	Copper resistance protein	24382..24560	178	C3R74_13315
7	PetA: Ubiquinol-cytochrome c reductase, iron-sulfur subunit	125547..126167	620	C3R74_04020
8	Fe^2+^ transport system protein B	117413..119761	2348	C3R74_06760
9	Sulphur reductase	51615..52115	500	C3R74_00250
10	Cytochrome C biogenesis protein ResB	242678..244522	1844	C3R74_04655
11	RES domain-containing protein	311917..312654	737	C3R74_05030
12	cycA: Cytochrome C	248549..249331	782	C3R74_04680
13	sdrA: Short-chain dehydrogenase	126198..127007	809	C3R74_04025
14	petB: Cytochrome b	124305..125519	1214	C3R74_04015
15	petC: Ubiquinol cytochrome C oxidoreductase	123581..124282	702	C3R74_04010
16	Ferric iron uptake transcriptional regulator	504255..504731	476	C3R74_06040
17	cysJ: Sulfite reductase subunit alpha	50107..51873	1766	C3R74_06360
18	cysI: Sulfite reductase	48426..50117	1691	C3R74_06355
19	cysH: Phosphoadenylyl-sulfate reductase	47681..48418	737	C3R74_06350
20	cysN: Sulfate adenylyltransferase	45394..46746	1352	C3R74_06340
21	cysD: Sulfate adenylyltransferase subunit CysD	46746..47681	935	C3R74_06345

Phylogenomic analysis performed with eight *Acidithiooxidans ferrooxidans* species and outgroup *Acidithiobacillus caldus* shows that isolates BY0502 and IO-2C both fall outside of the main cluster of closely related *A*. *ferrooxidans* strains (Supplementary Fig. 4).

RAST annotation revealed that genome of *Acidithiobacillus ferrooxidans* IO-2C comprised of 373 subsystems. The dormancy and sporulation subsystem featured persister cells enabling the bacterium to survive in extreme stressed conditions for longer period of time. The secondary metabolism subsystem is comprised of auxins. Sulphur metabolism is comprised of fifteen genes for inorganic sulphur assimilation and three for organic sulphur assimilation. The genome contains a variety of mobile elements in the form of integrons comprised of gene cassettes e.g. integron integrase Int, integron integrase IntI1, integron integrase IntI2, integron integrase IntI4, and integron integrase IntIPac, associated with transposons or plasmids promoting antibiotic resistance in strain. The respiration subsystem is comprised of 131 genes including 31 for biotin, 10 for ATP synthase, 39 for electron accepting reactions and 57 for electron donating reactions. In the virulence, disease and defence category 81 subsystems were found featuring bacteriocins (7), resistance to antibiotics and toxic compounds (61), copper homeostasis (7), cobalt-zinc-cadmium resistance (23), zinc resistance (2), mercuric reductase (1), mercury resistance operons (4), beta lactamases (2), multidrug resistance efflux pumps (8), resistance to fluoroquinolones (4), arsenic resistance (7) and copper tolerance (3). Four genes featuring metabolism of aromatic compounds were also identified in genome. Additionally, 22 genes for nitrogen fixation and 14 for ammonia assimilation were also found in genome.

### Pan genomic analysis

**Figure 5:**
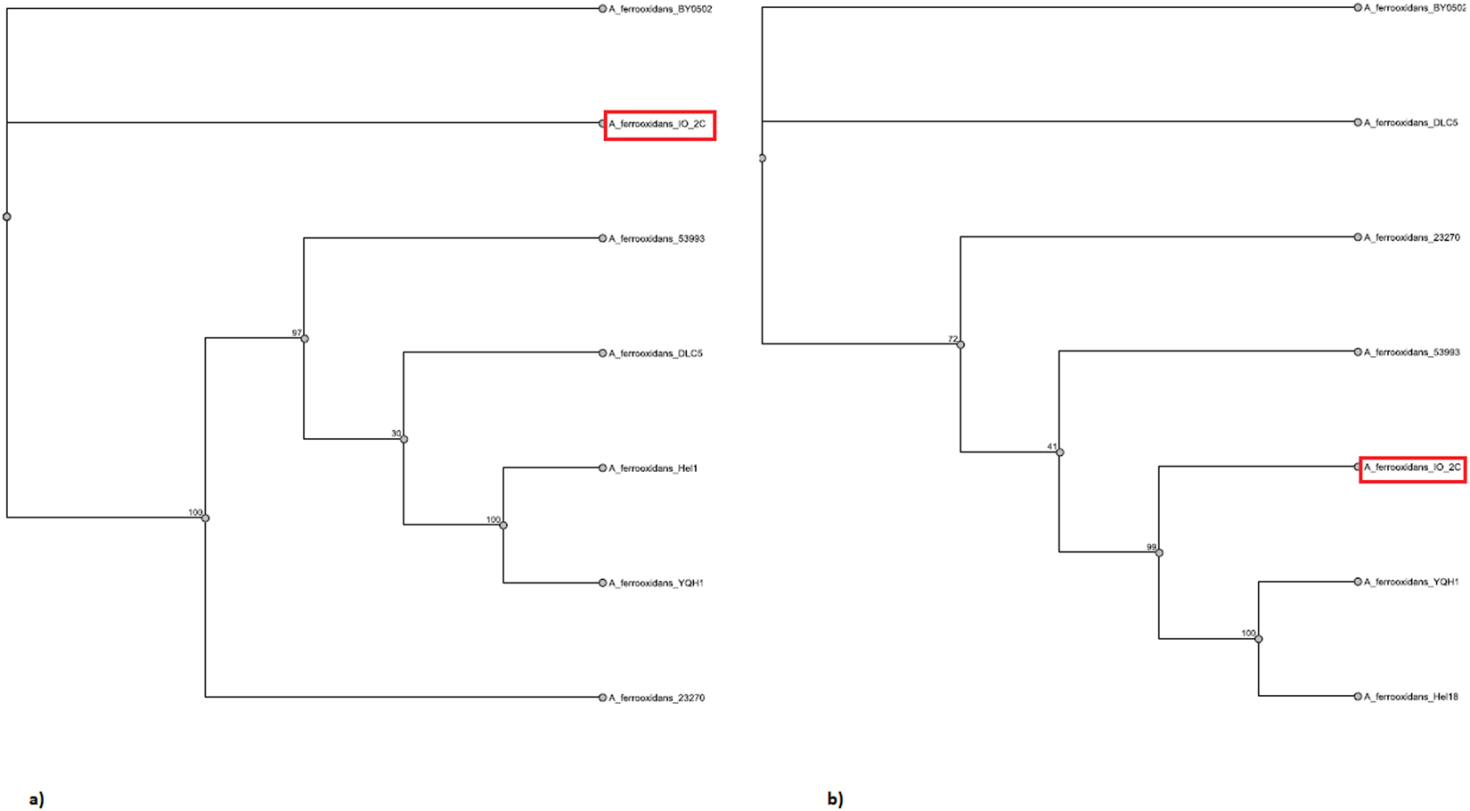
Phylogenetic analysis of the *Acidithiobacillus ferrooxidans* based on (**a)** pan and (**b)** core-genome similarity.

**Figure 6:**
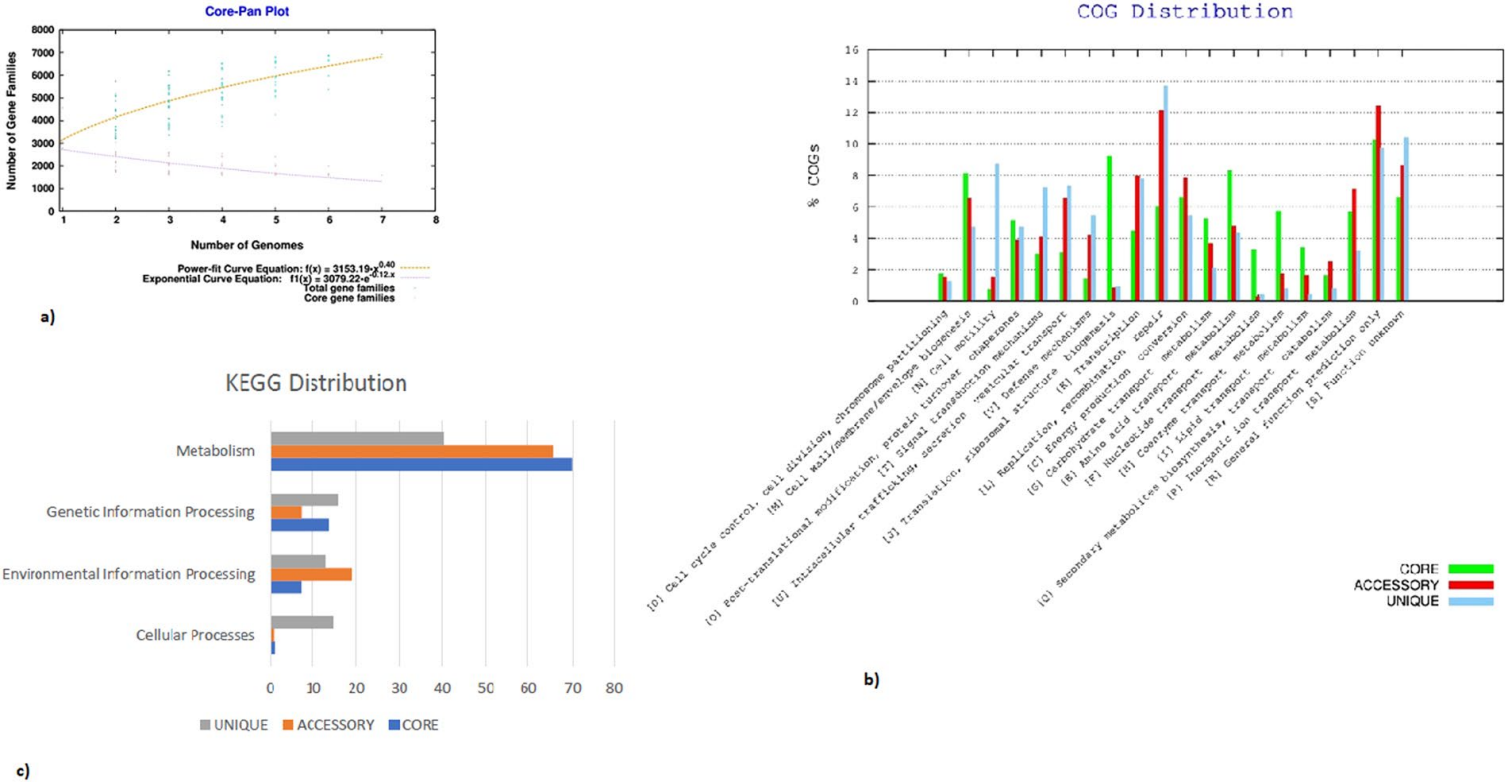
(**a**) Core-pan plot of genomes under study (**b)** COG and (**c)** KEGG distribution of the genes forming core, accessory and unique portion of genomes under study.

The pan-genome constitutes the repertoire of all the genes in a particular species including core genome (genes present in all strains), accessory genome (genes occur in two or more strains) and singletons (unique genes). This type of analysis has been used to determine the genetic diversity in a group of species pertaining to phenotypic traits, ecological adaptation, new host colonization, virulence and antibiotic resistance^12^. A total of seven genomes of *Acidithiobacillus ferrooxidans* were used for pan genome analysis (Table 2). Phylogenetic analysis with the pan genome showed *A*. *ferrooxidans* IO-2C and *A*. *ferrooxidans* BY0502 grouping together. However, in the core genome analysis *A*. *ferrooxidans* IO-2C was positioned together with two different strains, Hel18 and YQH1 (Fig. 5). Pan genome curve (Fig. 6a) indicated the openness of pan-genome which might be the consequence of horizontal gene transfer or natural evolution^13^. Most of the unique genes are related to information storage and processing while most of the core genes are associated to metabolic functions (Fig. 6b,c). Genes responsible for metabolic functions are relatively conserved throughout all the species; however, genes concerning genetic information, cellular processes and environmental information processing are more diverse and explicated heterogeneity due to ecological adaptation or lateral gene transfer. This genetic divergence of bacterial genomes is correlated with the presence of integrases and phage-associated genes which cause horizontal gene transfer and introduce new genes with novel functions. Recruitment of new genes and abandonment of few others in the *Acidithiobacillus ferrooxidans* genomes could be the strategy of niche adaptation^14^.

**Table 2:** Genomic properties of *Acidithiobacillus ferrooxidans* species used for pan-genome analysis along with strain IO-2C.

Serial no.	Bioproject ID	Organism name	No of Contigs	Genome size (bp)	G + C content (%)	No. of accessory genes	No. of unique genes	No. of exclusively absent genes
1	PRJNA53	*A*. *ferrooxidans* 23270	1	2,982,397	58.8	1190	257	1
2	PRJNA16689	*A*. *ferrooxidans* 53993	1	2,885,038	58.9	1122	86	6
3	PRJNA317193	*A*. *ferrooxidans* BY0502	295	2,976,670	56.8	613	944	399
4	PRJNA251676	*A*. *ferrooxidans* DLC5	2,090	4,184,217	57.6	1425	1547	23
5	PRJNA308169	*A*. *ferrooxidans* Hel18	123	3,109,160	58.6	1490	33	0
6	PRJNA294114	*A*. *ferrooxidans* YQH1	96	3,111,222	58.6	1491	36	1
**7**	**PRJNA432283**	***A***.***ferrooxidans*** **IO-2C**	**23**	**2**,**716**,**894**	**58**.**7**	**869**	**292**	**71**

Overall, majority of the genes involved in metabolic pathways are represented in the core genome of *Acidithiobacillus ferrooxidans*. The highest number of unique genes were shared by A. *ferrooxidans* DLC5 in pan-genome analysis (Supplementary Fig. 5).

### Variant analysis

Variant calling is an essential comparative genomic method that provides insights into organismal differences on nucleotide-level. It includes structural variants, single and multiple nucleotide polymorphisms (SNPs, MNPs), insertions and deletions (indels). It identifies genome coordinates with polymorphisms relative to a reference^15^ and allows predictions to be made as to the impact of variants that occur in genes. Here, we have found >75,000 variants in IO-2C when compared with each of the published genome sequences; of these variants, 287 are predicted to have a high impact on gene function resulting in frameshift mutations and gain/loss of stop codons. Table 3 describes the high-confidence single- and multiple-nucleotide polymorphisms as well as indels for all available *Acidithiobacillus* strains. As compared to other *A*. *ferrooxidans* strains, BY0502 showed the highest number of high-quality variants (103,486) with respect to strain IO-2C, but the high number of variants may be an artefact of the BY0502 assembly quality, since it is the most fragmented with 295 contigs. However, a study suggested that *A*. *ferrooxidans* BY0502 may have been misidentified, as it showed more relatedness with *A*. *ferrivora*ns on the basis of ANI and TETRA analysis^16^. Variants with a predicted high impact in *A*. *ferrooxidans* ATCC 23270 (frameshift, loss or gain of stop codon) and *A*. *ferrooxidans* ATCC 53993 are reported (Supplementary Table S1). Here we discuss 78 high-impact variants contained in genes of known function—an additional 209 (of the 287 total) high-impact variants are not discussed here as they are found in hypothetical genes with no predicted function. These sequence variants are expected to make some genes non-functional in IO-2C genome however, they are functional in all others including genes encoding integrase protein, thioredoxin, mobile element protein, Cytochrome d ubiquinol oxidase and NifX-associated protein (Supplementary Table S1).

**Table 3:** Summary of variants in IO-2C genome vs six reference strains.

	*A*. *ferrooxidans* ATCC 23270	*A*. *ferrooxidans* ATCC 53993	*A*. *ferrooxidans* BY0502	*A*. *ferrooxidans* HeI18	*A*. *ferrooxidans* YQH1
Total reads	8608053	8615233	8619333	8628253	8628509
Properly mapped reads	6888563 (80.02%)	6783099 (78.73%)	4715417 (54.71%)	6816839 (79.01%)	6827769 (79.13%)
Total reference bases	2982397	2885038	2946256	3100878	3098413
Mean base coverage	444.4	450.0	182.8	415.3	417.2
Raw FreeBayes variants	76508	79627	108939	80295	81114
High quality variants	75061	77796	103486	78319	79045

### Iron metabolism

The genome of IO-2C contain genes encoding a rusticyanin protein for the oxidation of Fe(II) to Fe(III). Protein assembly responsible for iron oxidation comprised of rusticyanin-related protein, Cytochrome C oxidase polypeptide III, Cytochrome C oxidase assembly factor, CoX10-CtaB, Cytochrome C oxidase polypeptide II, and Cytochrome C oxidase polypeptide I. A similar pattern of gene organization was observed in *Acidithiobacillus ferrooxidans* ATCC 23270 (Supplementary Fig. 6). Rusticyanin plays crucial role in the electron-transfer pathway from Fe (II) to oxygen. Levels of rusticyanin inside the cell are regulated by iron and sulfur. Rusicyanin is one of the major constituent of oxidative respiratory chain. Other components of the respiratory chain include cytochrome C, cytochrome A, and an iron-sulphur protein. Different studies have reported the expression of rusticyanin and their role in iron oxidation in *Acidithiobacillus ferrooxidans*^17,18^. These studies demonstrated that expression of the *rus* operon is induced and up-regulated in the presence of ferrous iron rather than sulphur.

**Figure 7:**
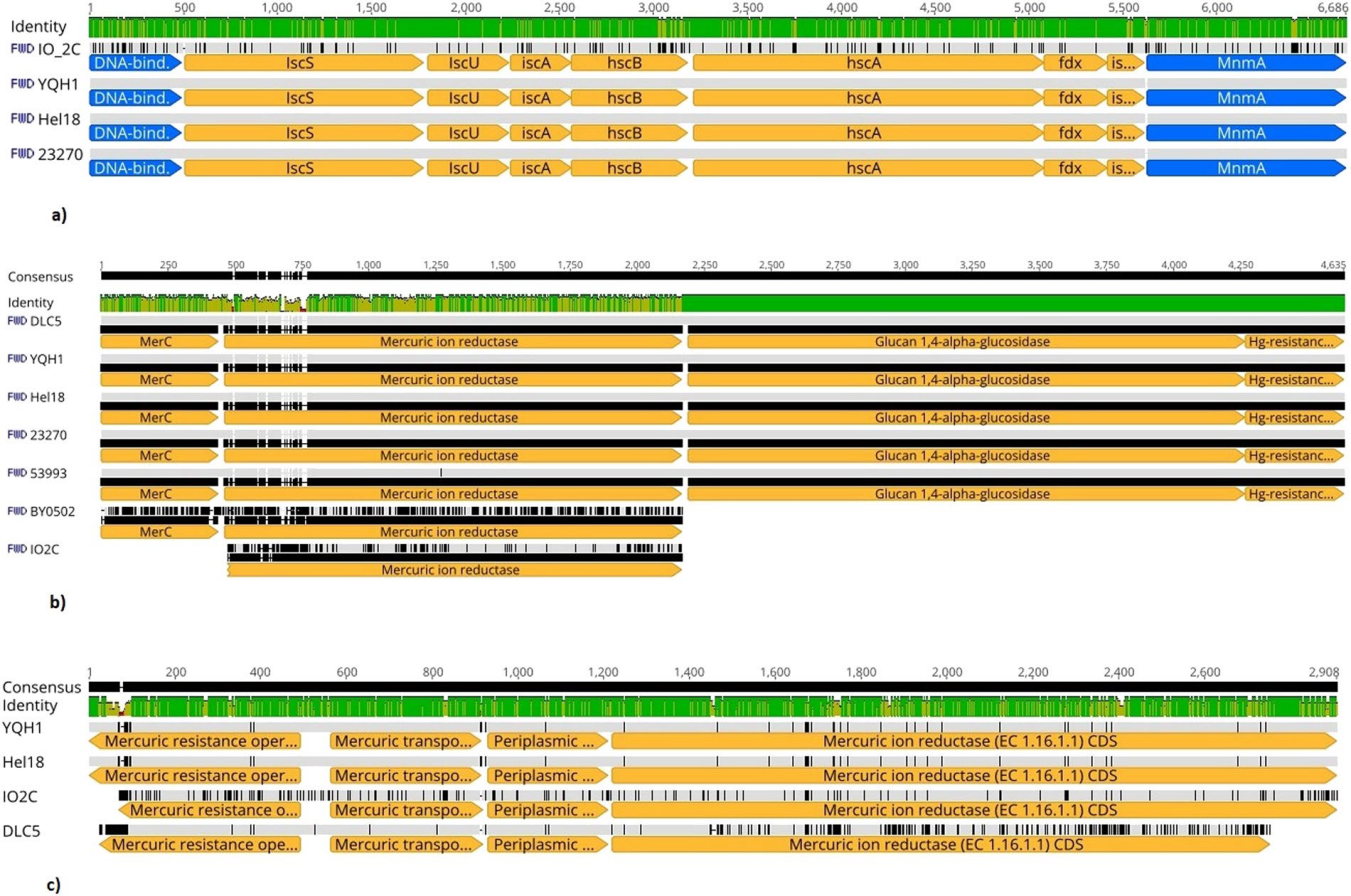
Multiple sequence alignments depicting (**a)** Gene apparatus encoding iron-sulfur cluster assembly (**b)** Mercury resistant gene cluster I (**c)** Mercury resistant gene cluster II in different *Acidithiobacillus ferrooxidans* species.

Genes encoding ferredoxin proteins were also detected in IO-2C genome. These are iron sulphur proteins abundant in biological redox cycles and play an essential role in the biosynthesis of Fe-S clusters. In bacteria, a cluster of eight genes encoding ferredoxin proteins is present in the “isc” operon. Ferredoxin protein containing [Fe_2_S_2_] cluster from *A*. *ferrooxidans* ATCC 23270 was expressed and cloned in *Escherichia coli* which was further purified and characterized by Zeng *et al*.^19^. When sequences encoding ferredoxin were aligned, IO-2C strain had 273 single base mutations plus a two bp insertion in the intergenic spacer between iscX and MnmA compared to the other three strains (275 bp different, total–95.9% identical) (Fig. 7a).

### Sulphur metabolism

The IO-2C genome contained a complete catalogue of genes responsible for uptake and regulation of sulfur metabolism including genes encode for sulphur reductase, sulphur transferases, cysteine desulphurase (NifS), ubiquinol-cytochrome c reductase (PetA) and peroxiredoxin involved in reduction of intracellular sulphur. A gene cluster i.e. IscA, IscU, and IscS, encoding protein for iron-sulphur (Fe-S) cluster assembly was also detected in IO-2C genome as previously identified in *A*. *ferrooxidans* ATCC23270. These genes are supposed to play part in the Fe-S centres formation and assimilation of sulphate into cysteine. They act as catalysts in metabolism and are crucial for electron transfer, nitrogen fixation and transcriptional control^20^.

### Heavy metal resistance

IO-2C genome also contained gene clusters for toxic heavy metal resistance like copper, mercury, and arsenic etc. Genes for mercury resistance comprised of two clusters. When compared with other strains, basically 5 of the seven strains had nearly identical merC-containing gene clusters, however, IO-2C and BY0502 were different. BY0502 lacked the mercury resistance operon regulator gene and IO-2C lacked both that and the merC gene (Fig. 7b). The other five strains were almost 100% identical for this region, with strain 53994 having a single G to C base pair substitution in the mercuric iron reductase gene that causes a non-synonymous substitution (Alanine to Glycine). Four of the strains, IO-2C, Hel18, DLC5, and YQH1 contained an additional gene cluster containing four mercury related genes. On one end of the cluster was merR, the mercury operon regulatory gene, and then in the opposite orientation were three consecutive mercury related genes: merT (mercury ion transport protein), Periplasmic mercury (II) binding protein, and a mercuric ion reductase–a different one from that found in the other mercuric gene cluster containing merC (Fig. 7c). The aligned four gene cluster for these four genes showed 81.8% sequence identity. BY0502 did not contain this gene cluster but did have a copy of merR with its copy of merC and a mercuric ion reductase. Strain 53993 contained a gene cluster with two copies of merC and merR each plus a mercuric ion reductase. Strain 23270 did not have anything resembling this gene cluster.

Determinants of copper resistance included ScsA, ScsB, ScsC, ScsD, CutA, CutC, CutF, CutE, CorC (Supplementary Fig. 7) whereas that of arsenic is encoded by arsD, arsA, arsB, arsC, ACR3, arsH, ArrA, ArrB, ArrS, ArsR2, CymA (Supplementary Fig. 8). Our findings of heavy metal resistance in IO-2C genome are supported by many other studies describing metal resistance in *Acidithiobacillus ferrooxidans*^21–23^. Metals become toxic when they accumulate in bacterial cell above regular physiological concentrations through metal transport systems. Inside the cell they form bond with enzymes functional groups, deter transport systems and disrupt cellular membranes^24^.

Because strain IO-2C is acidophilic, dwells in a metal-rich environment and is resistant to toxic metals it may be a suitable candidate for bioleaching of heavy metals. Although our analysis is preliminary and based primarily on bioinformatic predictions, our research group is further exploring the bioleaching capabilities of this bacterium *in vitro* to provide some tangible results.

## Conclusion

*Acidithiobacillus ferrooxidans* IO-2C contains a repertoire of genes required for living in a metal rich, acidic environment including a complement of genes for iron and sulphur metabolism, multidrug and heavy metal resistance, cell wall and capsule, virulence, disease and defence, potassium and phosphorous metabolism, membrane transport, protein metabolism, cell division and cell cycle, regulation and cell signalling, DNA metabolism, fatty acids, lipids, and isoprenoids, nitrogen metabolism, dormancy and sporulation, respiration, stress response, metabolism of aromatic compounds, and amino acids and derivatives. Moreover, IO-2C genome shows low gene conservation and high genetic variability as evident through OrthoANI and pan genome analysis, a finding further supported by variant analysis of the genome. Whole genome analysis has provided unique insights into the genetic and metabolic potential of *Acidithiobacillus ferrooxidans* IO-2C, suggesting its possible interaction in acid rich environment by preserving its bioenergetics through iron/sulphur redox processes and nitrogen fixation, formation of persister cells during unfavourable conditions, maintaining pH homeostasis and responding to anoxic environmental conditions. Genomic analysis presented in this study not only provided a coherent view of organism’s eco-physiology but also offered new insights for future experimental research signifying its potential role in bioremediation and biomining operations.

## Methods

### Sample collection, isolation and identification of bacterial strain

Site selected for sample collection was an acid seep area of the Texas Municipal Power Agency located in Carlos, Texas, USA at the latitude of 30.56 N and longitude of 96.06 E. The temperature of acid seep soil at the time of sample collection was about 33.5 °C and pH was 3.2. Soil was collected from surface layer of soil up to 6–8 inches, placed in sterilized zipper bags and transferred to Soil and Aquatic Microbiology Lab at Soil and Crop Sciences Department of Texas A&M University. For isolation of targeted microorganisms, the soil sample was enriched with 9K medium^25^ supplemented with 1% pyrite and incubated at 25 °C for fifteen days in rotary shaker at 120 rpm. About 50 µl of each enrichment was plated on ferrous tryptic soya agar plates^26^ and incubated at 25 °C. After incubation for one week, plates were observed for the presence of distinct brown bacterial colonies. Colonies were purified through single colony streaking by picking individual colonies onto ferrous tryptic soya agar (FTSA) plates. For genomic DNA extraction from purified bacterial cells grown on FTSA, Mo Bio UltraClean microbial DNA isolation kit was used and purified DNA was sent to Macrogen, Inc., Maryland, USA for 16S rRNA sequencing. Sequence obtained was submitted to NCBI GenBank to get accession number.

### Specific medium composition and growth conditions for bacterial strain

Ferrous tryptic soya broth (FeTSB) containing ammonium sulphate 0.9 g/l, magnesium sulphate 0.35 g/l, and tryptone soya broth 0.175 g/l was used for the growth of iron oxidizing bacterium. About 50 ml of medium was placed in each of 100 ml Erlenmeyer flasks. The pH of medium was adjusted to 2 ± 0.2. After autoclaving each flask was supplemented with 1% of any of iron substrate i.e. FeSO_4_ or (NH_4_)_2_Fe(SO_4_)_2_•6H_2_O and inoculated with fresh bacterial culture grown on ferrous tryptic soya agar. Flasks were placed in shaking incubator at 25 °C and 120 rpm for fifteen days.

### Quantification of ferrous iron oxidation

To determine the iron oxidation ability of bacterium, biomass was harvested from FeTSB medium through centrifugation at 10,000 g and cell free supernatant was collected after every two days. A ferrozine kit (Sigma Aldrich) was used to quantify the iron oxidation in cell free supernatant by following the standard protocol provided with the kit. Briefly, 100 µl of supernatant was placed in a 96- well flat bottom plate. For measuring Fe(II), 5 µl of iron assay buffer provided with the kit was added to each well. For measuring Fe(III), two sets of wells were set up for each sample. 5 µl of assay buffer was added in one set and 5 µl of Iron reducer provided with the kit to the other set of wells. Samples were mixed on a horizontal shaker and incubated at room temperature for thirty minutes. 100 µl of iron probe (provided in the kit) was added to each well containing samples, mixed and incubated at room temperature for an hour under dark conditions. Absorbance was measured after incubation at 593 nm. For preparing standards, 10 µl of the 100 mM iron standard provided in the kit was mixed with 990 µl of water to make 1 mM standard solution. Different volumes i.e. 1–100 µl of 1 mM standard solution were put into a 96 well plate to make different concentrations in nmole/well standards. Iron assay buffer was added in each well to dilute the sample up to 100 µl. Then 5 µl of iron reducer was added to each standard well and left for 30 minutes. Approximately 100 µl of iron probe was added to each standard well and incubated for an hour. Absorbance was measured after incubation at 593 nm and values obtained were used to plot standard curve (Supplementary Fig. 1). Concentration of Fe(II) and total iron was determined from the standard curve and that of Fe(III) was obtained by subtracting total iron i.e. sample with iron reducer from Fe(II) i.e. sample with assay buffer.

### Characterization of iron oxides bio-minerals produced by *Acidithiobacillus ferrooxidans* IO-2C

For extraction of bio-minerals, biomass along with minerals produced by bacterium in FeTSB medium were harvested after fifteen days of incubation. Medium was centrifuged at 10000 g for 20 minutes and supernatant was discarded. Pellets obtained were washed thrice with autoclaved distilled water to remove any media contents left and allowed to dry. The iron oxides in the pellets were characterized with X-ray diffraction (XRD), Fourier transform infrared spectroscopy (FTIR), and field-emission scanning electron microscope (FE-SEM). For the XRD analysis, each DI water washed pellet was dispersed in about 0.5 mL DI water in a micro-centrifuge tube, then the dispersion was transferred to and air dried on a zero-background quartz slide. The XRD pattern of each air-dried film on the slide was recorded in the 2° to 70° 2-theta range on a Bruker D8 ADVANCE X-ray diffractometer with a Cu Kα X-ray source operated at 40 kV and 40 mA. A step size of 0.05°, a dwell time of 3 seconds at each step, and a variable divergence slit programed to 12 mm radiation length were used in the XRD analysis. To eliminate the contribution of strong X-ray fluorescence from the iron oxides to the XRD intensity, an energy dispersive detector SolX (Bruker) was used to record the diffracted Cu Kα radiation only.

For the FTIR analysis, about one-quarter of the iron oxide film on the quartz slide was scratched off and transferred to the diamond crystal surface of a Universal Attenuated Total Reflection (ATR) accessory on the Perkin-Elmer System 100 Fourier transform infrared spectroscopy. Each FTIR spectrum was recorded in the wavenumber range of 4000–700 cm^−1^ with a resolution of 4 cm^−1^. A total of 32 scans were collected for each sample and averaged to obtain the spectrum.

For the SEM analysis, a few micro-liters of the pellet dispersion used in the XRD sample preparation was transferred to an SEM tab mounted on an SEM stub and dried under a 250-W heating lamp. The dried SEM specimen was coated with 4-nm Pt/Pd on a Cressington 208 HR sputter coater. Secondary electron and backscattered electron SEM images and the energy dispersive spectrum of interested particles were recorded on a FEI Quanta 600 FE-SEM system.

### Whole genome sequencing, assembly and annotation

A DNA sequence library was prepared using Nextera DNA Sample preparation kit (Illumina) following the manufacturer’s user guide. The concentration of DNA was evaluated using the Qubit® dsDNA HS Assay Kit (Life Technologies). Then 50 ng DNA was used to prepare the library. The sample underwent the simultaneous fragmentation and addition of adapter sequences. These adapters are utilized during a limited-cycle (5 cycles) PCR in which unique indices were added to the sample. Following the library preparation, the final concentration of the library was measured using the Qubit® dsDNA HS Assay Kit (Life Technologies), and the average library size was determined using the Agilent 2100 Bioanalyzer (Agilent Technologies). 10 pM of the library was clustered using the cBot (Illumina) and sequenced paired end for 500 cycles using the HiSeq 2500 system (Illumina). Whole genome sequence was assembled in NGEN (Biostar) version 12 and annotated with PATRIC^27^ (Pathosystems Resource Integration Center)/RAST^28^ (Rapid Annotation using Subsystem Technology) and NCBI Prokaryotic Genome Annotation^29^ pipelines.

### In-silico analysis of whole genome sequence

OrthoANI^30^ was used to calculate the average pairwise nucleotide identity (ANI) among the *Acidithiobacillus* genomes. A value of 95% and above is commonly interpreted as the species pairs belong to same genus. Sequences were aligned with MAFFT v7.388 (https://academic.oup.com/mbe/article/30/4/772/1073398) as implemented in Geneious^31^, and alignments were visualized in Geneious version 11.0.2. (https://www.geneious.com).

The phylogenetic tree was generated in PATRIC with eight *Acidithiobacillus ferrooxidans* species and outgroup *Acidithiobacillus caldus*. 100 single-copy genes were selected and aligned using MUSCLE^32^. The resulting alignment 42,274 amino acids (126,822 nucleotides) was used to generate a likelihood tree using RAxML^33^ under the GTRCAT substitution model; bootstrap support values are given on nodes where applicable.

A total of 7 *Acidithiobacillus* genomes were used for pan-genome analyses (6 from GenBank and the new genome from this study). BPGA (Bacterial Pan-genome Analysis tool) was used to estimate core, pan and species-specific genomes^12^. PATRIC was used to predict coding sequences for all seven genomes, and these amino acid sequences were used as input for BPGA. Initial clustering was done through Usearch^34^ algorithm and output processed into pan, core and accessory gene categories. The empirical power law equation f(x) = a.x^b and exponential equation f1(x) = c.e^(d.x) were used for extrapolation of the pan and core genome curves respectively. Presence or absence of genes/families was determined to infer species-specific gene families. For each new genome in the analysis pipeline 100 random permutations of genomes were carried out to avoid bias. Neighbour joining trees based on concatenated amino acid alignments and binary (presence/absence) pan-matrix were reconstructed.

Variant calling was performed on PATRIC^27^ with the raw paired-end Illumina reads used to assemble the IO-2C strain genome. The BWA-mem^35^ aligner was used to map reads against reference genomes and variants were called using FreeBayes. High-confidence single- and multiple-nucleotide polymorphisms, indels as well as variants with a predicted high impact are reported.

### Genome accession numbers

This Whole Genome Shotgun project of *Acidithiobacillus ferrooxidans* IO-2C has been deposited at DDBJ/ENA/GenBank (http://www.ncbi.nlm.nih.gov) under the accession PQJK00000000. The version described in this paper is version PQJK01000000.

## Supplementary information

Whole genome sequence analysis reveals high genetic variation of newly isolated Acidithiobacillus f

Dataset 1

## References

[1] Sharma, A., Parashar, D. & Satyanarayana, T. Acidophilic microbes: biology and applications. 215–241 (Springer International Publishing, 2016).

[2] Babu, P., Chandel, A. K. & Singh, O. V. Survival mechanisms of extremophiles. 9–23 (Springer, 2015).

[3] Nuñez H (2017). Molecular systematics of the genus Acidithiobacillus: insights into the phylogenetic structure and diversification of the taxon. Front. Microbiol..

[4] Yan L (2015). Draft genome sequence of *Acidithiobacillus ferrooxidans* YQH-1. Genom. Data.

[5] Yin H (2014). Whole-genome sequencing reveals novel insights into sulfur oxidation in the extremophile *Acidithiobacillus thiooxidans*. BMC Microbiol..

[6] Liljeqvist M, Rzhepishevska OI, Dopson M (2013). Gene identification and substrate regulation provide insights into sulfur accumulation during bioleaching with the psychrotolerant acidophile *Acidithiobacillus ferrivorans*. Appl. Environ. Microbiol..

[7] Horbaczewski, J. K. Mine-related and natural acid seeps at Gibbons Creek lignite mine, Texas. *28th Annual Surface Mine Reclamation Workshop*, *College Station*, *Texas*. (2007).

[8] Gadd GM (2010). Metals, minerals and microbes: Geomicrobiology and bioremediation. Microbiology.

[9] Valdés J (2008). *Acidithiobacillus ferrooxidans* metabolism: from genome sequence to industrial applications. BMC Genomics.

[10] Nazari B, Jorjani E, Hani H, Manafi Z, Riahi A (2014). Formation of jarosite and its effect on important ions for *Acidithiobacillus ferrooxidans* bacteria. Trans. Nonferrous Met. Soc. China.

[11] Tran TT (2017). Comparative genome analysis provides insights into both the lifestyle of *Acidithiobacillus ferrivorans* strain cf27 and the chimeric nature of the iron-oxidizing Acidithiobacilli genomes. Front. Microbiol..

[12] Chaudhari NM, Gupta VK, Dutta C (2016). BPGA-an ultra-fast pan-genome analysis pipeline. Sci. Rep..

[13] Basharat Z, Yasmin A, He T, Tong Y (2018). Genome sequencing and analysis of *Alcaligenes faecalis subsp*. *phenolicus* MB207. Sci. Rep..

[14] Zhang X, Liu X, Yang F, Chen L (2018). Pan-genome analysis links the hereditary variation of *Leptospirillum ferriphilum* with its evolutionary adaptation. Front. Microbiol..

[15] DePristo MA (2011). A framework for variation discovery and genotyping using next-generation DNA sequencing data. Nature Genet..

[16] González C, Lazcano M, Valdés J, Holmes DS (2016). Bioinformatic analyses of unique (orphan) core genes of the genus Acidithiobacillus: functional inferences and use as molecular probes for genomic and metagenomic/transcriptomic interrogation. Front. Microbiol..

[17] Sasaki K (2003). Respiratory isozyme, two types of rusticyanin of *Acidithiobacillus ferrooxidans*. Biosci. Biotechnol. Biochem..

[18] Yarzabal A, Appia-Ayme C, Ratouchniak J, Bonnefoy V (2004). Regulation of the expression of the *Acidithiobacillus ferrooxidans* rus operon encoding two cytochromes c, a cytochrome oxidase and rusticyanin. Microbiology.

[19] Zeng J, Huang X, Liu Y, Liu J, Qiu G (2007). Expression, purification, and characterization of a [Fe_2_S_2_] cluster containing ferredoxin from *Acidithiobacillus ferrooxidans*. Curr. Microbiol..

[20] Valdés J, Veloso F, Jedlicki E, Holmes D (2003). Metabolic reconstruction of sulfur assimilation in the extremophile *Acidithiobacillus ferrooxidans* based on genome analysis. BMC Genomics.

[21] Sugio T (2001). Cytochrome c oxidase purified from a mercury-resistant strain of *Acidithiobacillus ferrooxidans* volatilizes mercury. J. Biosci. Bioeng..

[22] Almarcegui RJ (2014). New copper resistance determinants in the extremophile *Acidithiobacillus ferrooxidans*: a quantitative proteomic analysis. J. Proteome Res..

[23] Xu Y, Yang M, Yao T, Xiong H (2014). Isolation, identification and arsenic-resistance of *Acidithiobacillus ferrooxidans* HX3 producing schwertmannite. J. Environ. Sci..

[24] Dopson M, Baker-Austin C, Koppineedi PR, Bond PL (2003). Growth in sulfidic mineral environments: metal resistance mechanisms in acidophilic micro-organisms. Microbiology.

[25] Li J (2016). Sulfur transformation in microbially mediated pyrite oxidation by *Acidithiobacillus ferrooxidans*: Insights from X-ray photoelectron spectroscopy-based quantitative depth profiling. Geomicrobiol. J..

[26] Mahmoud KK, Leduc LG, Ferroni GD (2005). Detection of *Acidithiobacillus ferrooxidans* in acid mine drainage environments using fluorescent *in situ* hybridization (FISH). J. Microbiol. Methods.

[27] Wattam AR (2017). Improvements to PATRIC, the all-bacterial bioinformatics database and analysis resource center. Nucleic Acids Res..

[28] Aziz RK (2008). The RAST Server: rapid annotations using subsystems technology. BMC Genomics.

[29] Tatusova T (2016). NCBI prokaryotic genome annotation pipeline. Nucleic Acids Res..

[30] Lee I, Kim YO, Park SC, Chun J (2016). OrthoANI: an improved algorithm and software for calculating average nucleotide identity. Int. J. Syst. Evol. Microbiol..

[31] Kearse M (2012). Geneious Basic: an integrated and extendable desktop software platform for the organization and analysis of sequence data. Bioinformatics.

[32] Edgar RC (2004). MUSCLE: a multiple sequence alignment method with reduced time and space complexity. BMC Bioinformatics.

[33] Stamatakis A (2014). RAxML version 8: a tool for phylogenetic analysis and post-analysis of large phylogenies. Bioinformatics.

[34] Edgar RC (2010). Search and clustering orders of magnitude faster than BLAST. Bioinformatics.

[35] Li, H. Aligning sequence reads, clone sequences and assembly contigs with BWA-MEM. *arXiv Preprint arXiv*, 1303.3997 (2013).

