# Supplementary material for: Whole genome sequence analysis reveals high genetic variation of newly isolated Acidithiobacillus ferrooxidans IO-2C
Source: Sci Rep. 2019 Sep 10;9:13049. doi: 10.1038/s41598-019-49213-x (PMC6736930; doi:10.1038/s41598-019-49213-x)
Supplement: Supplementary file 1 — Whole genome sequence analysis reveals high genetic variation of newly isolated Acidithiobacillus f [file 41598_2019_49213_MOESM1_ESM.pdf]

Whole genome sequence analysis reveals high genetic variation of newly isolated *Acidithiobacillus ferrooxidans* IO-2C

Anila Fariq[1, 2], John C Blazier[3], Azra Yasmin[1], Terry J. Gentry[2], Youjun Deng[2]

1 Microbiology & Biotechnology Research Lab, Department of Biotechnology,

Fatima Jinnah Women University, Rawalpindi, 46000, Pakistan

2 Department of Soil and Crop Sciences, Texas A&M University, College Station, TX, 77843, USA

3 Texas A&M Institute of Genome Sciences and Society, Texas A&M University, College Station, TX, 77843, USA

***Corresponding author**

Email: azrayasmin@fjwu.edu.pk

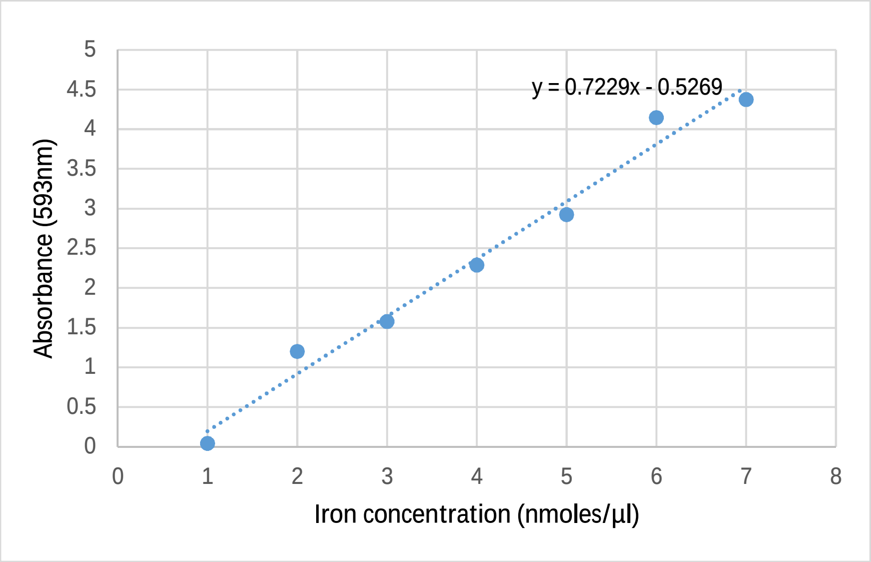

Supplementary Fig. 1. Iron Standard Curve

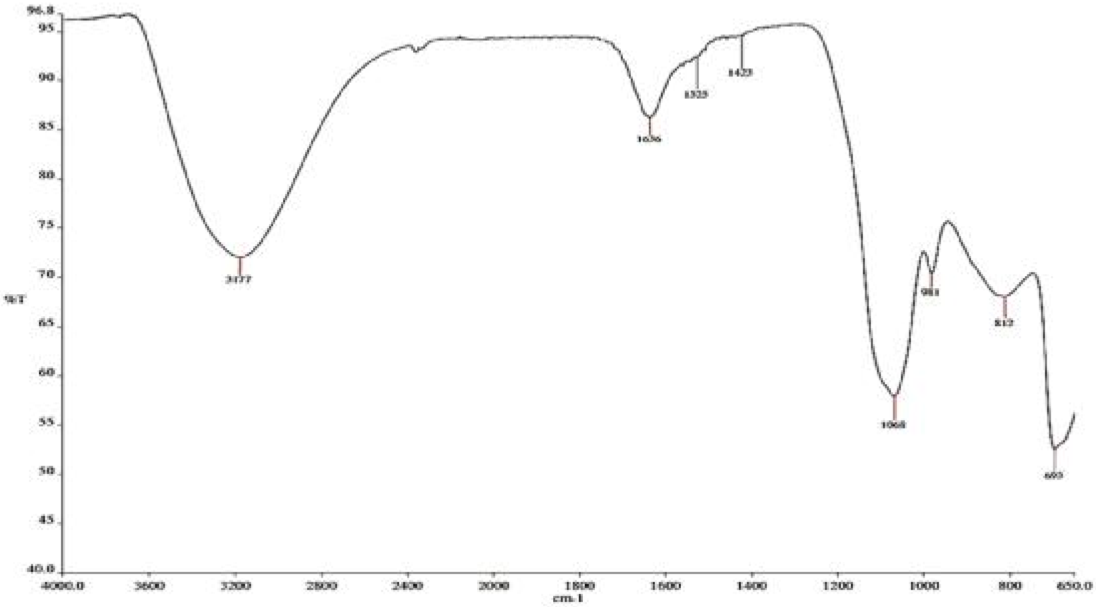

a)

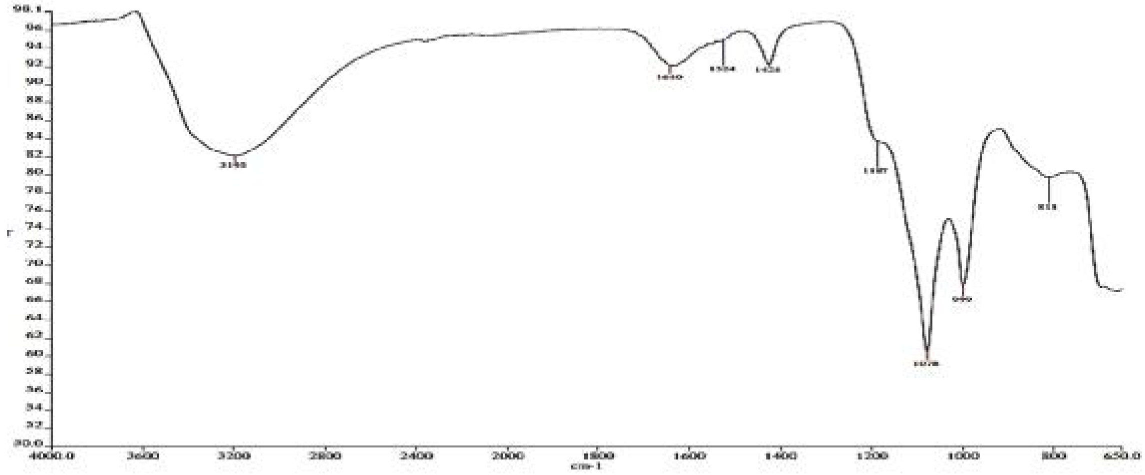

b)

Supplementary Fig. 2. IR spectra of iron oxides produced by *Acidithiobacillus ferrooxidans* IO-2C using **a)** $FeSO_4$ **b)** $Fe(NH_4)_2SO_4$ as substrates

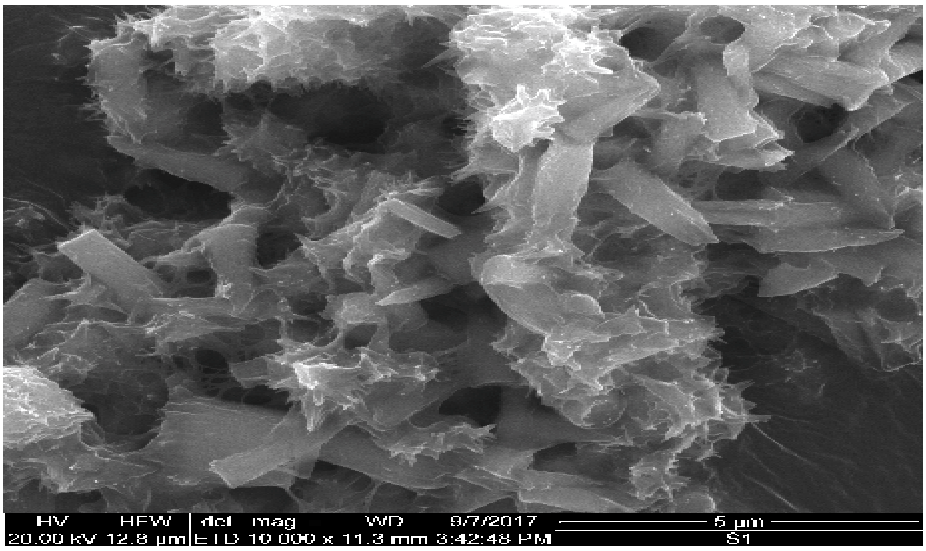

a)

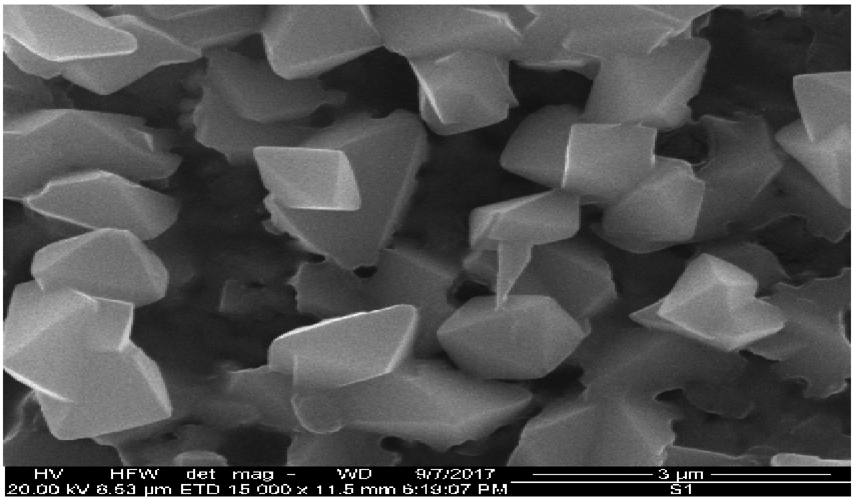

b)

Supplementary Fig. 3. SEM images of iron oxides produced by *Acidithiobacillus ferrooxidans* IO-2C using **a)** $FeSO_4$ **b)** $Fe(NH_4)_2SO_4$ as substrates

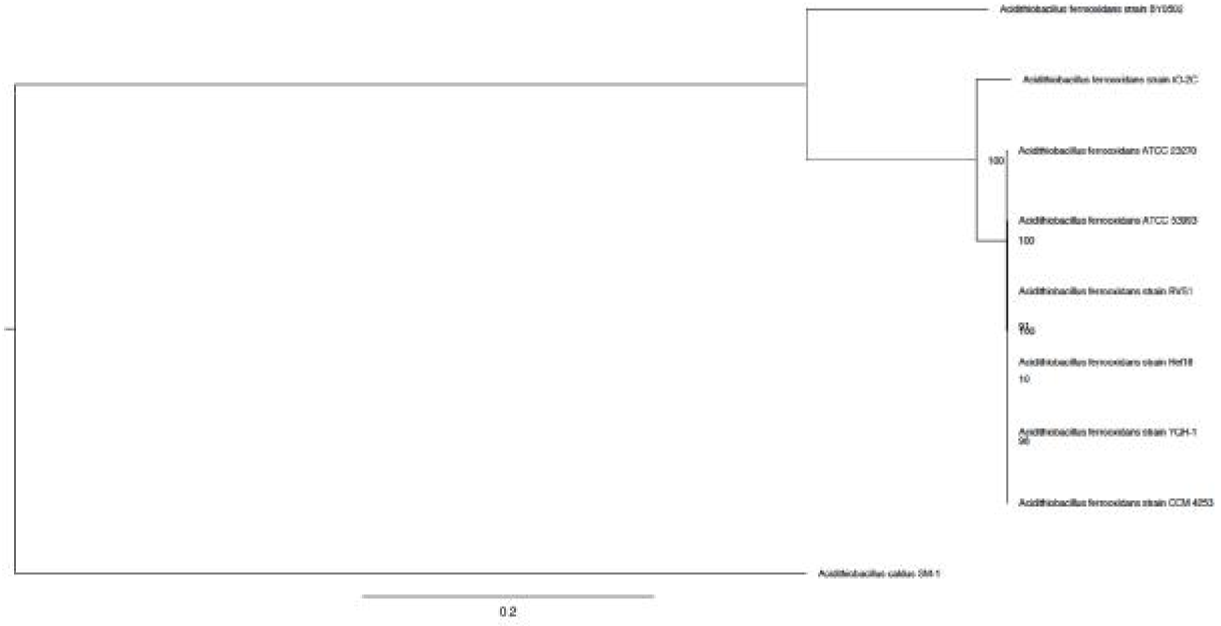

Supplementary Fig. 4. Phylogenetic tree of eight *Acidithiobacillus ferrooxidans species* and outgroup *Acidithiobacillus caldus*.

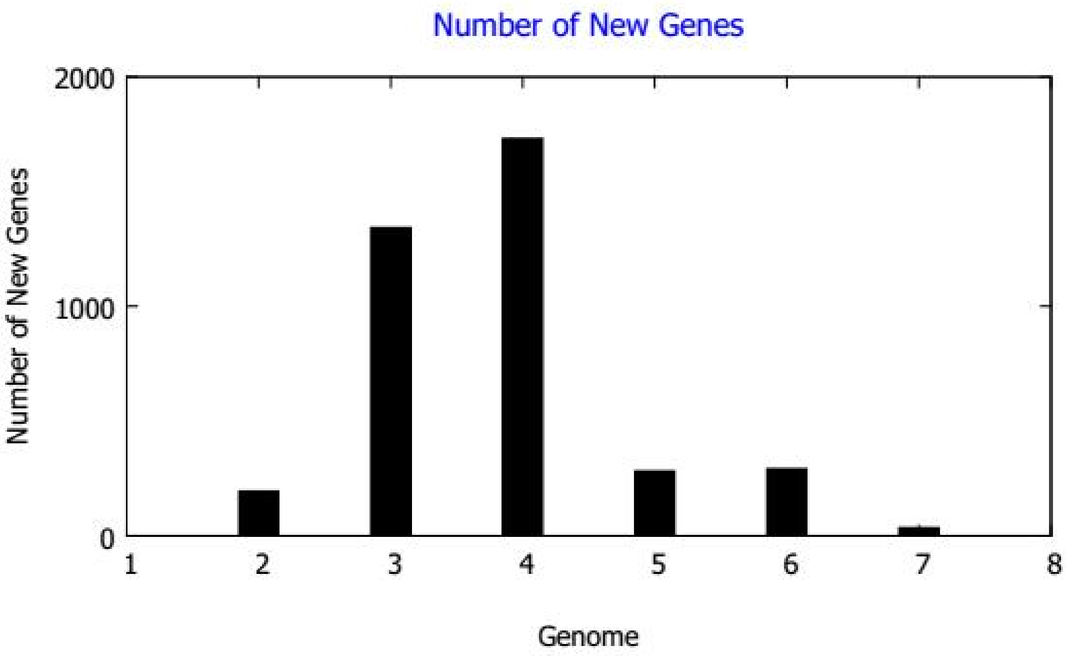

Supplementary Fig. 5. Number of new genes contributing to pan-genome

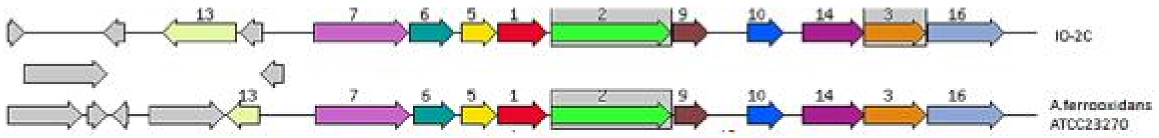

Supplementary Fig. 6. Assembly of iron oxidizing proteins in IO-2C and *A. ferrooxidans* ATCC23270. 10 rusticyanin related protein, 9 Cytochrome C oxidase polypeptide III, 14 Cytochrome C oxidase assembly factor, 3 CoX10-CtaB, 1 Cytochrome C oxidase polypeptide II, and 2 Cytochrome C oxidase polypeptide I

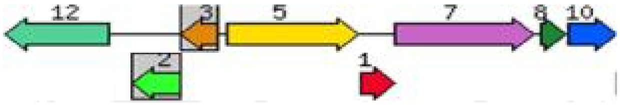

Supplementary Fig. 7. Organization of arsenic resistance determinants in IO-2C genome. 1 Arsenic resistance protein ArsH, 2 Arsenate reductase, 3 Transcriptional regulators, ArsR family, 5 Arsenic efflux pump protein, 7 Signal recognition particle, subunit Ffh SRP54, 8 SSU ribosomal protein, 10 16S rRNA processing protein 12 Membrane anchor protein YbhG

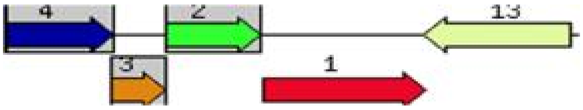

Supplementary Fig. 8. Determinants of copper resistance in IO-2C genome. 1 copper homeostasis protein CutE, 2 Magnesium and cobalt efflux protein CorC, 3 Metal dependent hydrolase, 4 Predicted ATPase, 13 TIdE protein, 16 Zinc ABC transporter
